# Stenotrophomonas maltophilia Bacteremia Associated With Severe COVID-19: Successful Treatment With Appropriate Antimicrobial Therapy

**DOI:** 10.7759/cureus.21750

**Published:** 2022-01-30

**Authors:** Masaatsu Kuwahara, Mitsuki Noma, Takuya Sunakawa, Kunihiro Shirai, Keisuke Kohama, Atsushi Miyawaki, Jun-ichi Hirata

**Affiliations:** 1 Emergency and Critical Care Medicine, Hyogo College of Medicine, Nishinomiya, JPN

**Keywords:** trimethoprim–sulfamethoxazole, stenotrophomonas maltophilia, covid-19, central venous catheter infection, covid: bacteremia

## Abstract

*Stenotrophomonas maltophilia, *an opportunistic pathogen, can cause bacteremia in immunocompromised and debilitated patients. A 50-year-old man with severe coronavirus disease 2019 (COVID-19) was admitted to our hospital’s intensive care unit where he underwent extracorporeal membrane oxygenation and ventilatory support. On day 25, he developed *S. maltophilia* bacteremia originating from an indwelling central venous catheter. After confirming susceptibility, trimethoprim-sulfamethoxazole (80 mg/400 mg) was administered thrice daily. Following improvement, he was weaned from ventilation, recovered sufficiently, and was discharged on day 53. To the best of our knowledge, this is the first report of a patient recovering after antimicrobial treatment for *S. maltophilia* bacteremia associated with severe COVID-19.

## Introduction

*Stenotrophomonas (Xanthomonas) maltophilia,* a ubiquitous, aerobic, nonfermentative, multidrug-resistant, Gram-negative, rod-shaped bacterium, is closely related to the genus *Pseudomonas* and can be an opportunistic pathogen, especially in hospitalized patients [1].

Pneumonia and bacteremia are the most common manifestations of* S. maltophilia* infection; most cases of *S. maltophilia* bacteremia are associated with an indwelling catheter [2]. Studies have demonstrated that 73% of 207 patients receiving oncologic treatment who had indwelling central venous catheters and *S. maltophilia* bloodstream infections had catheter-related infections, 22% had secondary infections (primarily pulmonary in origin), and 5% had primary infections unrelated to the catheter [3]. Because *S. maltophilia* is a multidrug-resistant organism, there are limited antibiotic options.

*Stenotrophomonas maltophilia *infections in severely immunocompromised and debilitated individuals are associated with high morbidity and mortality, with overall mortality rates ranging from 21% to 69% [4,5]. Intensive care unit admission and delay in effective treatment are independent risk factors for mortality [6].

The incidence of opportunistic infections like *S. maltophilia *may have increased during the coronavirus disease 2019 (COVID-19) pandemic because of the increased number of prolonged intensive care unit stays for extracorporeal membrane oxygenation (ECMO) and ventilation. Herein, we report the case of a patient with severe COVID-19 and *S. maltophilia* bacteremia who was successfully treated at our hospital and discharged.

## Case presentation

The patient was a 50-year-old man with a body mass index of 28 kg/m2 and no relevant medical history. He was a past smoker (20 cigarettes/day for 15 years) with no drinking or COVID-19 vaccination history. After being diagnosed with COVID-19 pneumonia by polymerase chain reaction, he was referred to our hospital and admitted to the intensive care unit because it was difficult to maintain oxygenation with normal oxygen therapy and ventilator management became necessary. *Stenotrophomonas maltophilia* was detected in blood cultures (two bottles/two sets) on day 25. Below, we describe his course of treatment before and after day 25 of hospitalization.

General condition

On the second day after admission to our hospital, the patient was found to have poor oxygenation and was subsequently intubated and placed on ventilation. On the seventh day of hospitalization, ECMO was initiated due to difficulty maintaining oxygenation despite ventilatory support. The patient’s oxygenation improved, enabling weaning from ECMO on day 23 of hospitalization, at which stage he still required mechanical ventilation.

Drugs

Tocilizumab (8 mg/kg/day) was administered on the first and second days of hospitalization for severe COVID-19. Steroids were administered as follows: dexamethasone (6 mg/day) for the first six days, methylprednisolone (250 mg) for three days from days seven to nine, methylprednisolone (80 mg) for nine days from days 10 to 18, methylprednisolone (40 mg) for five days from day 19 to 23, and, finally, prednisolone (20 mg/day), which was still being administered on day 25.

Antimicrobial therapy comprised ceftriaxone (2 g) once daily for four days from day four to seven as empirical therapy for ventilator-associated pneumonia, which was also treated empirically with cefozopran hydrochloride (1 g) twice daily for two days on days 13 and 14. After the detection of extended-spectrum β-lactamase-producing *Klebsiella pneumoniae* in the patient’s sputum, meropenem (1 g) was administered thrice daily for eight days from days 14 to 21. The patient was de-escalated to cefmetazole sodium (1 g) thrice daily for four days from days 22 to 25.

*Stenotrophomonas maltophilia *was detected in the sputum on the fourth day of hospitalization, after that, it continued to be detected. However, because there was no associated worsening of respiratory symptoms, the patient was considered to be a carrier. Thus, no treatment was initiated for *S. maltophilia* infection at this time.

Course from day 25

On day 25, the patient had a fever of 38.4 °C, and *S. maltophilia* was detected in blood cultures taken to identify the source of infection. This organism was also detected in the central venous catheter tip submitted for analysis on the same day. Thus, bacteremia due to catheter infection was diagnosed. Because the organism was strongly sensitive to trimethoprim-sulfamethoxazole (TMP-SMX) (minimum inhibitory concentration ≤ 1), TMP-SMX was started at 80 mg/400 mg thrice daily (the standard treatment for *S. maltophilia* infection). The patient’s general condition improved, and he was successfully weaned from the ventilator and extubated on day 28. Trimethoprim-sulfamethoxazolewas administered intravenously for 14 days from days 25 to 38. Subsequently, the patient recovered sufficiently for discharge on day 53 of hospitalization.

## Discussion

We present a rare case of bacteremia caused by *S. maltophilia* during severe COVID-19 infection. Only one case of bacteremia caused by *S. maltophilia *has been reported in a patient with severe COVID-19, and that patient ultimately died [7]. In the present case, *S. maltophilia* bacteremia during severe COVID-19 was successfully treated with antimicrobial therapy.

Risk factors associated with *S. maltophilia* infection include intensive care unit admission, human immunodeficiency virus (HIV) infection, malignancy, cystic fibrosis, neutropenia, mechanical ventilation, indwelling central venous catheter, recent surgery, trauma, and history of broad-spectrum antibiotic treatment [8]. In the present case, the following risk factors were present: intensive care unit admission, indwelling central venous catheter, and history of broad-spectrum antibiotic treatment. Most cases of *S. maltophilia* bacteremia are associated with an indwelling catheter, which was also the case with our patient.

Treatment of severe COVID-19 inevitably requires intensive care unit admission. Additionally, broad-spectrum antibiotic therapy for secondary infection is frequently needed. Furthermore, these patients require many other drugs, including sedatives, analgesics, and hypertensive agents, making the insertion of a central venous catheter essential. In addition, long-term ventilation, tracheostomy, and ECMO are all more frequently required by patients with severe COVID-19 than by those with other diseases that require mechanical ventilation.

The administration of dexamethasone has been shown to reduce mortality in patients with COVID-19 [9] and is, thus, the current standard of care. However, this may result in steroid-induced immunosuppression. It is, therefore, necessary to consider *S. maltophilia* as a possible cause of bacteremia in patients with severe COVID-19.

*Stenotrophomonas maltophilia* infections should be treated promptly with antibiotics because any delay of appropriate treatment can lead to significant mortality. Given that *S. maltophilia *is a multidrug-resistant organism, antibiotic options are limited. Trimethoprim-sulfamethoxazole is the antibiotic combination of choice because of its reliable in vivo activity against *S. maltophilia* [10]. In the present case, we confirmed the organism’s susceptibility to TMP-SMX; prompt administration of this antibiotic combination led to a good outcome. Notably, our patient was about 10 years younger than the one reported by Pek et al. [7], and improvements in the treatment of COVID-19 since they treated their patient may have contributed to our patient’s good outcome.

Nevertheless, the present case provides some points for reflection. In particular, *S. maltophilia *was detected by sputum culture on the fourth day of hospitalization. At this point, we judged that he was only a carrier, rather than having an infection and accordingly did not administer any treatment. However, it may have been preferable to initiate TMP-SMX treatment when bacteremia was first suspected.

## Conclusions

It is necessary to consider *S. maltophilia* as a possible cause of bacteremia in patients with severe COVID-19. It may be better to start treatment with TMP-SMX when bacteremia is suspected, especially for *S. maltophilia* carriers. In conclusion, to the best of our knowledge, this is the first case report of a patient with severe COVID-19 and *S. maltophilia* bacteremia who was successfully treated and discharged home.
